# Electrical Status Epilepticus in Sleep (ESES) in an Elderly Adult: A Case Report

**DOI:** 10.7759/cureus.26372

**Published:** 2022-06-27

**Authors:** Audrey Nath, Elliott Whitworth, Donnie Bretz, Daniel Davila-Williams, Lori McIntosh

**Affiliations:** 1 Neurology, University of Texas Medical Branch, Galveston, USA; 2 Neurology, University of Kentucky College of Medicine, Lexington, USA; 3 Neurology, Owensboro Health Regional Hospital, Owensboro, USA; 4 Pediatric Neurology, Texas Children's Hospital - Baylor College of Medicine, Houston, USA

**Keywords:** epilepsy, older adults, elderly, electrical status epilepticus in sleep, eses

## Abstract

Electrical status epilepticus in sleep (ESES) is a pattern of continuous spikes seen in electroencephalography (EEG) and may be associated with neuropsychological deficits in children. This EEG pattern has not previously been reported in older adults. In this case report, a 66-year-old woman with post-traumatic epilepsy presented to the emergency department following a breakthrough seizure. Her EEG exhibited a striking pattern of continuous spikes during sleep that stopped abruptly with wakefulness, which is characteristic of the ESES phenomenon. This patient had triggers for a breakthrough seizure including subtherapeutic seizure medication levels, exposure to flashing lights, and iatrogenic hyperthyroidism, but none of these triggers have been known to cause selectively continuous spikes during sleep on EEG. This finding suggests that the phenomenon of ESES may persist into older adulthood.

## Introduction

Electrical status epilepticus in sleep (ESES) describes an electroencephalographic (EEG) pattern of continuous spike and slow-wave activity during non-rapid eye movement (non-REM) sleep seen in children [1]. It is hypothesized that the pathophysiology of this electrographic abnormality may be related to thalamic dysfunction [2]. In children, ESES appears in clinical syndromes including seizure activity and neurocognitive regression [3].

While ESES is a well-described electrographic phenomenon in children, reports of ESES in adults are rare. There is one case report of a 27-year-old woman with a history of epilepsy and developmental delay who was found to have an ESES pattern on EEG; in this case, the etiology was suspected to be related to a cerebral folate deficiency [4]. There is another case report of an ESES pattern seen in a 21-year-old which then persisted in the same patient at 25 years of age [5]. However, there are no reported cases of ESES in older adults.

In this present study, we present a case of an ESES pattern on EEG in an elderly adult, which to our knowledge has not been previously described.

## Case presentation

A 66-year-old woman with a history of post-traumatic epilepsy and hypothyroidism was brought to the emergency department following a breakthrough seizure at home. She had been prescribed phenytoin for her epilepsy for over 40 years, but her husband reported that she had not been consistently taking her seizure medication or seen an outpatient neurologist in years. She was watching television and saw flashing lights on the screen. After seeing the flashing lights, she experienced generalized tonic stiffening with an atonic component and hit her head on a table in the process. Following the generalized tonic stiffening, she had generalized clonic movements with noted urinary incontinence. This episode of status epilepticus lasted a total of 27 minutes before stopping without pharmacologic intervention. The patient did not have any memory of the event.

During workup in the emergency department, it was noted her phenytoin level was subtherapeutic, at 6.3 mcg/mL (therapeutic range 10.0-20.0 mcg/mL). She had a head CT performed with no acute intracranial abnormalities; no previous head imaging was available for comparison. Lactic acid was elevated at 3.9 mmol/L. Thyroid function was abnormal with a high T3 uptake of 51%, low thyroid-stimulating hormone level of 0.03 mU/L, and T4 level within normal limits. Urinalysis detected a small amount of blood and 3+ bacteriuria, with no leukocytes or nitrates. Complete blood count, vitamin B12, folate, and chemistries were unremarkable. Blood alcohol testing and urine drug screening were both negative.

The patient’s epilepsy reportedly began during childhood. When she was between the ages of 12 and 14 years, she sustained a traumatic brain injury from falling off a horse. One year after the accident, she began having seizures. Per history, she had been taking phenytoin since that time. Her last reported convulsive seizure with impaired awareness was 41 years ago, although it was not clear if she had been having any focal seizures with intact awareness. There is no reported history of developmental delays. The remainder of her medical history is non-contributory.

**Figure 1:**
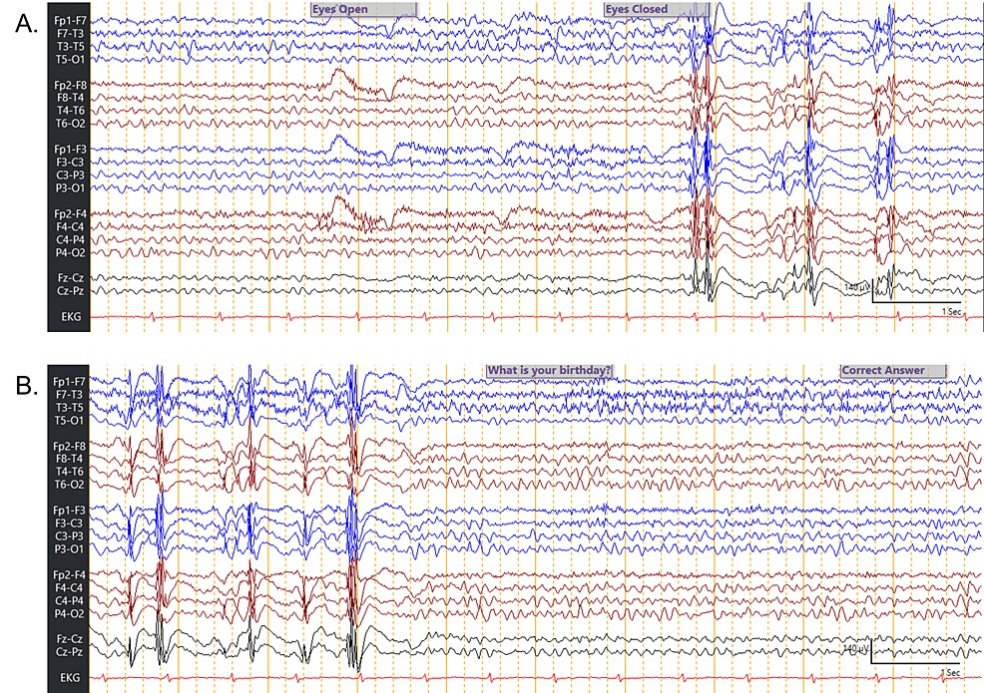
The awake and asleep EEG of the 66-year-old patient A: Awake EEG of the patient transitioning to continuous spikes with eye closure, B: Pattern of continuous spikes in sleep abruptly transitioning to awake pattern with technologist instructing the patient to answer a question EEG: Electroencephalography

A routine EEG was performed the next day (Figure 1). When the patient was asked a question, she was aroused, and the EEG background had a clear bilateral posterior dominant rhythm of 8 to 9 Hz, with occasional multifocal spikes. After answering questions from the technologist, the patient appeared to doze off. Immediately after falling asleep, the EEG background changed to a continuous pattern of high-amplitude multifocal spikes and slow-wave discharges. There were no previous EEGs available for comparison. 

To help treat the ESES pattern, as would be recommended with an inpatient child with this pattern [6], the addition of valproic acid was offered; the patient declined to initiate this medication. The patient has not been to an outpatient or inpatient encounter since this event for a follow-up on her condition. 

## Discussion

In this case, we describe a case of an ESES pattern on EEG in an older adult. Within the span of a brief routine EEG, there was a striking pattern of continuous multifocal spikes during light sleep. These continuous spike-wave discharges were then interrupted by a relatively normal awake background when the patient was asked to respond to questions. As such, the marked increase of spikes during sleep was compared to wakefulness, with spikes present in over 50% of the sleep recording, which is consistent with ESES as per the European Academy of Neurology (EAN), European Sleep Research Society (ESRS), and The International League Against Epilepsy (ILAE)-Europe consensus review [7].

The noted multifocal pattern of spikes during sleep may be seen with ESES, which may consist of a multifocal or generalized distribution of spikes [8]. Magnetoencephalography was not available to better clarify the localization of epileptic foci. 

The patient in this case had several likely triggers for her breakthrough seizure, but none with any known association with ESES. She had a noted subtherapeutic phenytoin level that may be a trigger for breakthrough seizures but it has not been known to cause ESES specifically. Her episode of status epilepticus occurred while watching flashing lights on television, which has been a noted form of photic stimulation that may trigger seizures in susceptible individuals [9]. Photic stimulation, however, has not been known to trigger a continuous pattern of spikes during sleep but wakefulness. Furthermore, she was noted to have a history of hypothyroidism and an iatrogenic hyperthyroid state while in the emergency department. While thyroid hormone dysfunction may have a theoretical role in epileptogenesis [10], there have been no reported cases of hyperthyroidism triggering ESES. In one other case of ESES in an adult patient, there was a noted cerebral folate deficiency [4]; our patient did not exhibit any anemia or folate deficiency.

Given that the known triggers for the patient’s breakthrough status epilepticus are unlikely to have caused continuous spikes during sleep, and that ESES is generally a pattern seen in children, it may be more likely that this patient has had ESES since childhood. While ESES is generally thought to be an age-related phenomenon in children, two cases have been described in adults in their 20s (Table 1) [4,5].

**Table 1:** Case reports of adults with ESES

Author	Age of patient	Suspected etiology
Thome et al. [4]	27 years	Cerebral folate deficiency
Mariotti et al. [5]	25 years	Persistence of ESES pattern from earlier childhood

Based on the known previous cases of ESES in adulthood, this pattern may persist into adulthood in some patients. By extrapolation, it appears possible for this electrographic pattern to persist until later adulthood, as in our patient.

While ESES is known to be associated with neurocognitive dysfunction in children, in our case, there were no reported developmental delays; however, in an elderly adult, recollection of the details of school performance or earlier childhood may be limited. It is possible that there were neuropsychological deficits after the onset of her epilepsy, coinciding with the timing of the earliest potential appearance of ESES in this patient.

## Conclusions

In summary, this case of a continuous spike pattern in sleep in an EEG of an elderly adult with epilepsy suggests a possibility of ESES persisting into older adulthood. To better understand the progression of this electrographic pattern, longitudinal studies of children with known ESES with repeat EEG studies into their early and late adulthood would be helpful in the future.
